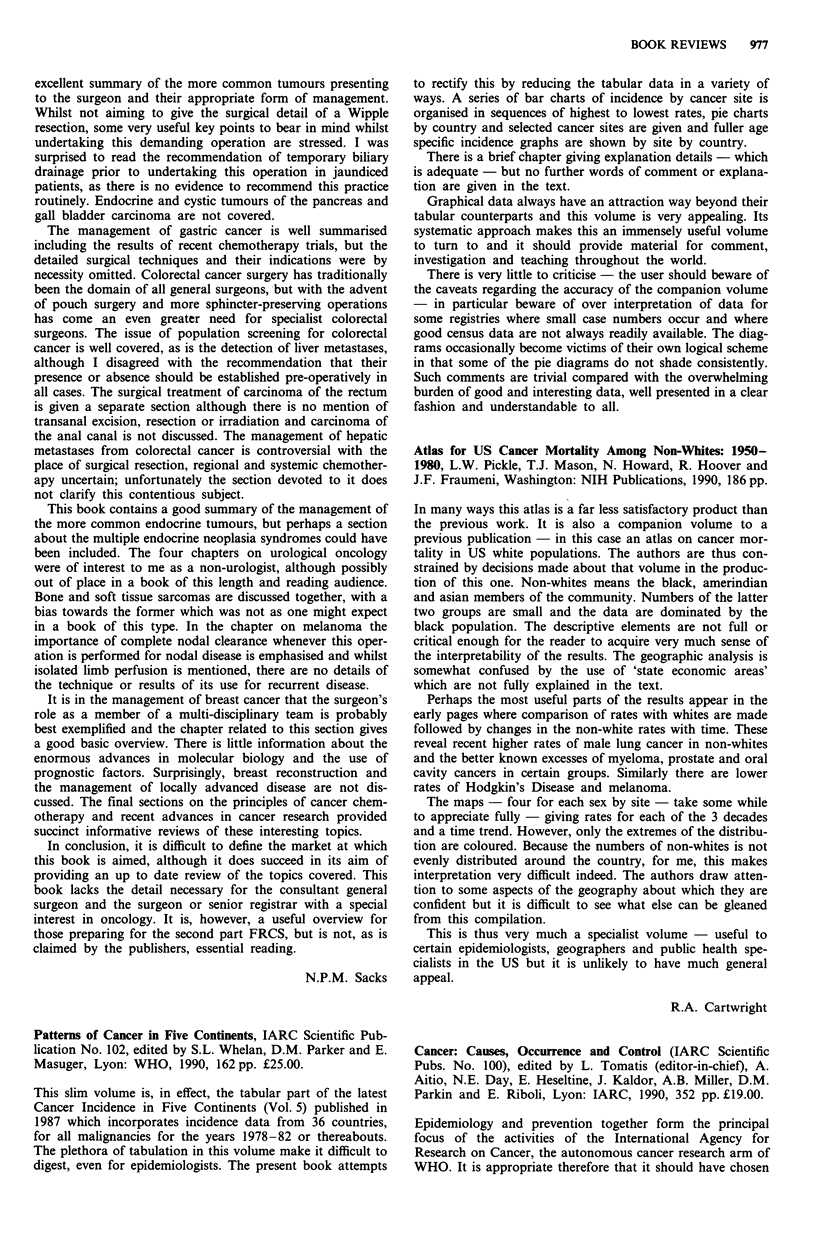# Atlas for US Cancer Mortality Among Non-Whites: 1950-1980

**Published:** 1991-11

**Authors:** R.A. Cartwright


**Atlas for US Cancer Mortality Among Non-Whites: 1950–1980**, L.W. Pickle, T.J. Mason, N. Howard, R. Hoover and J.F. Fraumeni, Washington: NIH Publications, 1990, 186 pp.

In many ways this atlas is a far less satisfactory product than the previous work. It is also a companion volume to a previous publication — in this case an atlas on cancer mortality in US white populations. The authors are thus constrained by decisions made about that volume in the production of this one. Non-whites means the black, amerindian and asian members of the community. Numbers of the latter two groups are small and the data are dominated by the black population. The descriptive elements are not full or critical enough for the reader to acquire very much sense of the interpretability of the results. The geographic analysis is somewhat confused by the use of 'state economic areas' which are not fully explained in the text.

Perhaps the most useful parts of the results appear in the early pages where comparison of rates with whites are made followed by changes in the non-white rates with time. These reveal recent higher rates of male lung cancer in non-whites and the better known excesses of myeloma, prostate and oral cavity cancers in certain groups. Similarly there are lower rates of Hodgkin's Disease and melanoma.

The maps — four for each sex by site — take some while to appreciate fully — giving rates for each of the 3 decades and a time trend. However, only the extremes of the distribution are coloured. Because the numbers of non-whites is not evenly distributed around the country, for me, this makes interpretation very difficult indeed. The authors draw attention to some aspects of the geography about which they are confident but it is difficult to see what else can be gleaned from this compilation.

This is thus very much a specialist volume — useful to certain epidemiologists, geographers and public health specialists in the US but it is unlikely to have much general appeal.

R.A. Cartwright